# Molluscs—A ticking microbial bomb

**DOI:** 10.3389/fmicb.2022.1061223

**Published:** 2023-01-09

**Authors:** Agnieszka Kijewska, Aleksandra Koroza, Katarzyna Grudlewska-Buda, Tomasz Kijewski, Natalia Wiktorczyk-Kapischke, Katarzyna Zorena, Krzysztof Skowron

**Affiliations:** ^1^Department of Immunobiology and Environmental Microbiology, Institute of Maritime and Tropical Medicine, Medical University of Gdańsk, Gdańsk, Poland; ^2^Department of Climate and Ocean Research and Education Laboratory, Institute of Oceanology Polish Academy of Science, Sopot, Poland; ^3^Department of Microbiology, Nicolaus Copernicus University in Toruń, Ludwik Rydygier Collegium Medicum, Bydgoszcz, Poland

**Keywords:** molluscs, bivalves, bacteria, antibiotic-resistance, genes, foodborne diseases, zoonoses, aquaculture

## Abstract

Bivalve shellfish consumption (ark shells, clams, cockles, and oysters) has increased over the last decades. Following this trend, infectious disease outbreaks associated with their consumption have been reported more frequently. Molluscs are a diverse group of organisms found wild and farmed. They are common on our tables, but unfortunately, despite their great taste, they can also pose a threat as a potential vector for numerous species of pathogenic microorganisms. Clams, in particular, might be filled with pathogens because of their filter-feeding diet. This specific way of feeding favors the accumulation of excessive amounts of pathogenic microorganisms like *Vibrio* spp., including *Vibrio cholerae* and *V. parahaemolyticus, Pseudomonas aeruginosa*, *Escherichia coli*, *Arcobacter* spp., and fecal coliforms, and intestinal enterococci. The problems of pathogen dissemination and disease outbreaks caused by exogenous bacteria in many geographical regions quickly became an unwanted effect of globalized food supply chains, global climate change, and natural pathogen transmission dynamics. Moreover, some pathogens like *Shewanella* spp., with high zoonotic potential, are spreading worldwide along with food transport. These bacteria, contained in food, are also responsible for the potential transmission of antibiotic-resistance genes to species belonging to the human microbiota. Finally, they end up in wastewater, thus colonizing new areas, which enables them to introduce new antibiotic-resistance genes (ARG) into the environment and extend the existing spectrum of ARGs already present in local biomes. Foodborne pathogens require modern methods of detection. Similarly, detecting ARGs is necessary to prevent resistance dissemination in new environments, thus preventing future outbreaks, which could threaten associated consumers and workers in the food processing industry.

## Graphical Abstract

Bivalve aquacultures are frequently treated with antibiotics for the protection of farmed animals. Extensive antibiotic treatment, by the presence of antibiotics in the water, may generate bacterial strains carrying the antibiotic-resistance (AR) genes in Mobile Genetic Elements (MGE). MGEs transmitted among marine bacteria may also be passed to pathogens in farmed animals and cause foodborne outbreaks. New AR strains might spread in a different environment through water, infected animals and supply chains of seafood and consumers. One of the most known spreading mechanisms is passing AR strains through faeces removed to the water environment. The circulation mechanism of AR genes leads to the need to use new antibiotics, which will probably stop being effective after a certain period of use. 
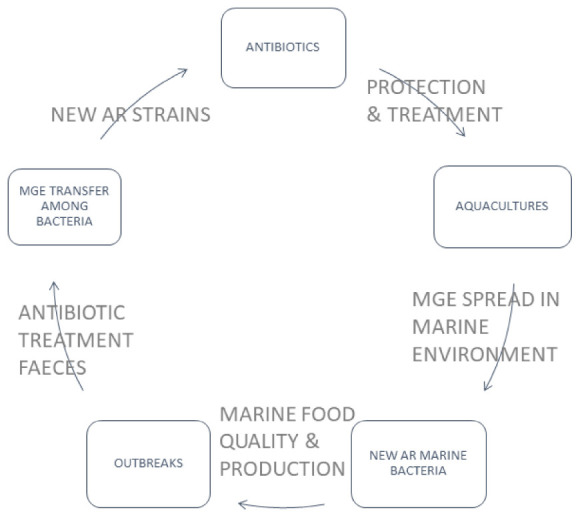

## 1. Introduction

Various bivalve molluscs (phylum Mollusca, class Bivalvia) constitute a significant commodity in fisheries and aquacultures but are also crucial to preserving our ecosystem’s complexity and function. All over the world, bivalve production has increased significantly in recent years. In 2014, global aquaculture production reached 101.1 million tonnes (live weight); in 2018, global aquaculture production, including aquatic plants, was 114.5 million tonnes, with an estimated USD 263 billion (Food and Agriculture Organization [FAO], 2020). In 2022, FAO (Food and Agriculture Organization of United Nations) reported that between 1992 and 2022, world aquaculture production of molluscs increased by about 70 million tonnes (Food and Agriculture Organization [FAO], 2022).

Aquaculture worldwide experienced significant development from the beginning of the 1980s. Increased awareness of the consequences of the long-term, intensive fishing for the wild fish stocks raised an ecological concern which intensified the development of bivalve aquacultures, now widely spread along worldwide coasts. In consequence, the European Union (EU) report about aquaculture displays that EU aquaculture accounts for about 20% of fish and shellfish supply in the EU and directly employs about 70,000 people. In 2020, more than 45% of aquaculture production was shellfish (European Market Observatory for Fisheries and Aquaculture Products [EUMOFA], 2022). According to information published by European Market Observatory for Fisheries and Aquaculture Products [EUMOFA] (2022), the EU’s organic aquaculture production has increased by 60% in 5 years (2015–2020), mainly due to a rise in organic mussel production. The Netherlands, Italy, Germany, Ireland, Denmark, France, Spain, and Bulgaria noted a significant increase in organic mussel production. Organic mussel production accounted for 41,936 tonnes in 2020 (10% of EU mussel production), compared to 18,379 tonnes in 2015. Only production of exclusive oysters increased in France, the most significant European producer, from 900 tonnes in 2018 to 3,220 tonnes in 2020. These numbers represent the scale of production and growth of shellfish share in the European seafood market (European Market Observatory for Fisheries and Aquaculture Products [EUMOFA], 2022).

The bivalves are very attractive food rich in protein (Wright et al., 2018). Usually, shellfish are prepared, cooked, and marinated, but locally, they are also sold fresh. The microbiota in molluscs depends on regions and may change according to climatic changes, salinity, pollution, and other factors. Gut microorganisms play a significant role in the absorption of essential nutrients and produce the energy they need throughout the day, and without gut microbiomes, digesting and breaking down compound molecules from food cannot be accomplished (Ramamurthy et al., 2014; Diwan et al., 2022). Previously, phenotypic characterization, including culture media (also quantitative assessment) and biochemical tests, were used to identify different species that make up the microbiome.

Molluscs such as oysters, mussels, scallops, cockles, and clams might filter and concentrate environmental microorganisms in their tissues (Wright et al., 2018; Bighiu et al., 2019). These microorganisms may still be active when the molluscs are eaten raw. Bivalve molluscs, including blue mussels (*Mytilus edulis*), great scallops (*Pecten maximus*), flat oysters (*Ostrea edulis*), horse mussels (*Modiolus modiolus*), and carpet shells (*Mya arenaria*), are suspension feeders and actively filter and retain particles from their surrounding water, including free-living or particle-bound bacteria (Kueh and Chan, 1985; Romalde et al., 2014; Hsu and Lee, 2015; Grevskott et al., 2016, 2017; Zannella et al., 2017; Mudadu et al., 2021). Bivalves and the blue mussel (*Mytilus edulis*) are successfully used as indicator organisms for marine pollution monitoring. The general assumption is that the mussel appears to be an appropriate sentinel organism because of its global distribution of large and accessible populations, its large size and sedentary adulthood, its tolerance to diverse environmental conditions, the ventilation of large volumes of water for nutrition, respiration, and excretion (Krieger et al., 1981) and its ability to accumulate numerous contaminants (Moy and Walday, 1996).

Musella et al. (2020) noticed that alongside their ecological importance, mussels have a relevant economic value as a species of interest in aquaculture and, at the same time, have long been employed in the biomonitoring of environmental quality in coastal areas. That factor is one of the most interesting because any change in the environment, including the anthropogenic transformation, may result in changes in the microbiota (Lopez-Joven et al., 2018), also showing the increasing problem of antibiotic-resistance genes (ARG) presence in the marine environment and, in consequence, in marine animals.

Antibiotic-resistance (AR) is a serious and growing global health threat. One of the significant sources of AR is the massive use of the broad spectrum of antibiotics in livestock production. Antibiotics are used there for the following purposes: Therapeutic—to treat sick animals; prophylactic—administrated at sub-therapeutic exposure concentrations to prevent disease; metaphylactic—in mass medication to eliminate or minimize an expected outbreak, and as growth, promoters to improve the growth rate and the food conversion (Pepi and Focardi, 2021). According to Chen et al. (2020), only a few countries/international institutions systematically confine the latter purpose due to AR spreading in the environment.

This monitoring of the current status of AR genes in the marine environment is essential due to the possible role of marine bacteria as the reservoir of AR genes and because the spreading of AR genes can increase the number of potentially resistant human and animal pathogens (Ramamurthy et al., 2014; Chen et al., 2020).

## 2. Anthropogenic impact on marine microbiota

**FIGURE 1:**
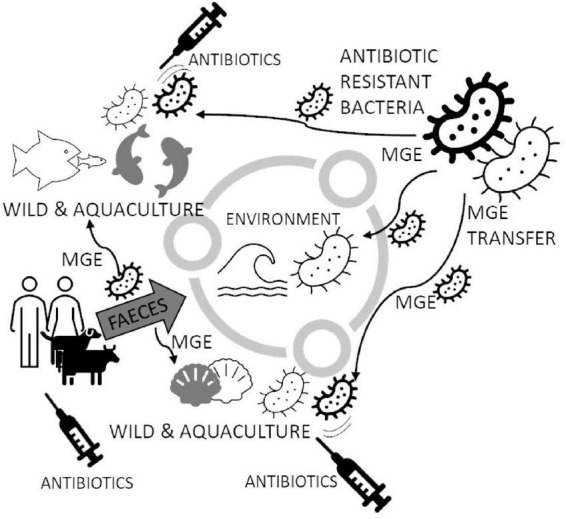
Circulating Mobile Genetic Elements (MGE) may increase Antibiotic Resistant (AR) marine bacterial species in the marine environment. Bacteria in the marine environment may spread resistance genes to other species, especially when antibiotics appear (like in fish aquacultures and bivalves’ larval hatcheries). The spread of MGE generates new strains of bacteria capable of successfully breaking out and becoming resistant to popular antibiotics. Antibiotics are delivered to water also with feces from coastlines, which are usually densely populated. The figure is prepared based on literature (Krieger et al., 1981; Kueh and Chan, 1985; Moy and Walday, 1996; Islam and Tanaka, 2004; Li et al., 2012; Weis, 2014; Hsu and Lee, 2015; Beaudry et al., 2016; Zannella et al., 2017; Chiesa et al., 2018; Kolm et al., 2018; Lopez-Joven et al., 2018; MacFadden et al., 2018; Bondarczuk and Piotrowska-Seget, 2019; Fresia et al., 2019; Hoegh-Guldberg et al., 2019; Pinsky et al., 2019; Chen et al., 2020; Grabowska et al., 2020; Martinez-Albores et al., 2020; Musella et al., 2020; Preena et al., 2020; Verga et al., 2020; Albano et al., 2021; Mudadu et al., 2021; Pepi and Focardi, 2021; Bagi and Skogerbø, 2022; Baker et al., 2022; Kamermans et al., 2022).

Marine pollution is a global and complex problem. Chemical compounds (including antibiotics) and pathogens, mainly introduced to the environment through human activities, appear to be several risks to biota, human health and ecosystems (Weis, 2014; Kolm et al., 2018; Verga et al., 2020). Sewage contains diverse polluting agents that directly and indirectly affect ecosystems and organisms (Islam and Tanaka, 2004; Wright et al., 2018; Bagi and Skogerbø, 2022; Diwan et al., 2022). In many coastal areas, submarine sewage outfalls are used to dispose of partially or fully treated wastewater. In addition to microbial diversity, antibiotics released to sewage may cause significant changes in gene abundances, especially virulence and ARGs, which have been identified in anthropogenically impacted marine and freshwater environments (Bondarczuk and Piotrowska-Seget, 2019; Fresia et al., 2019). In particular, sewage discharges into shallow coastal and subtidal environments may favor eutrophication processes, leading to water and sediment quality deterioration and, finally, decrease or affect biodiversity, with related ecological problems (Grabowska et al., 2020; Figure 1). Water contamination can result in increased numbers of pathogenic bacteria in mussels (Martinez-Albores et al., 2020) because most of the bivalves feed on suspended phytoplankton caught from water by pumping water across the gills. Laboratory results show that bacteria concentration in mussels *Dreissena polymorpha* is 132 times higher than in surrounding water, persisting several days after peak exposition (Martinez-Albores et al., 2020). This way, the pathogenic bacteria’s level increases compared to contaminated water (Bighiu et al., 2019).

The marine environment’s microbiological quality depends on many factors, but aquacultures might be considered one of the sources of pollutants. An exponential growth of aquaculture production in recent decades, which increased from less than 1 million tonnes in the 1950s to over 1225 million tonnes in 2020 (FAO), brought this problem into the Ocean (Preena et al., 2020). Bivalves are a significant part of aquaculture production, reaching nearly 18 million tonnes, including ornamental seashells and pearls farming. Aquaculture products’ microbiological quality depends on the water quality and other factors, including seafood preparation to consumption. These factors might represent human and animal sources of microorganisms, contaminating, among other, sedentary molluscs (Wright et al., 2018; Bighiu et al., 2019; Diwan et al., 2022). Also, as a consequence of the use of antibiotics in aquaculture, antibiotic-resistance is induced in the surrounding bacteria in the column water, sediment, and fish-associated bacterial strains (Pepi and Focardi, 2021).

Climate change may directly affect the molluscs’ aquacultures by increasing the water temperature above their optimal tolerance limits (Pinsky et al., 2019; Albano et al., 2021), supporting hypoxia and bacterial growth and their resistance to antimicrobials. A positive correlation between higher temperatures and raised antimicrobial resistance of *Staphylococcus aureus, Escherichia coli*, and *Klebsiella pneumoniae* was described by MacFadden et al. (2018). The climate anomalies also may lead to decreasing primary food production because of suboptimal living conditions led to low oxygen saturation and lowered immunocompetence, which increases the risk of infections (Beaudry et al., 2016; Kamermans et al., 2022). Also, climate change, including high air and water temperatures, rainfalls and droughts, may support the spread of food- and waterborne pathogens and, in effect, probably the increased number of outbreaks in the human population (Pepi and Focardi, 2021; Baker et al., 2022). Higher temperatures also favor spoilage processes and require earlier attempts to stop the degradation of the biochemical and microbiological processes of animal tissue, given the fact that for many regions, including the Mediterranean and central Europe, food availability is estimated to be reduced following a 2°C temperature increase (Hoegh-Guldberg et al., 2019).

### 2.1. Spreading of antibiotics in water

The continuous presence of antibiotics in bivalves may affect animals and bacterial microbiota living in and on water animals (Figure 1). Chiesa et al. (2018), using an HPLC–MS/MS multiclass method, developed a method for the determination of 29 antibiotics belonging to eight different chemical classes (penicillin, quinolones, tetracyclines, sulphonamides, macrolides, lincosamides, cephalosporins, and amphenicols), in mussels and clams, both wild and farmed, collected from various geographic areas of the world and, particularly, Italy. In 100 samples of *Meretrix lyrata* (the lyrate Asiatic hard clam), *M. meretrix* (the Asiatic hard clam), *Venerupis decussata* (the cross-cut carpet shell), *V. philippinarum* (the Manila clam), *Paphia textile* (the carpet clam), and *Chamelea gallina* (the Venus clam), they found four representatives of tetracycline group (49.45 ng g^–1^ tetracycline, 125.03 ng g^–1^ oxytetracycline, 60.45 ng g^–1^ doxycycline, and 77.48 ng g^–1^ chlortetracycline) in one pool of farmed clams obtained from the Italian side of the North Adriatic Sea. Considering the annual Per capita consumption of 0.33 kg clams (European Commission, data), the daily consumption is 0.91 g; the result of the multiplication of this value by the sum of the concentrations of the four tetracyclines (312.41 ng g^–1^) found in the clam sample of North Adriatic Sea, is 0.29 μg per day. Molluscs from Bohai Sea (11 species of commercially available molluscs *Crassostrea talienwhanensis, Chlamys farreri, Amussium, Scapharca subcrenata, Meretrix meretrix L., Mactra veneriformis, Mactra chinesis, Mya arenaria, Neverita didyma, Rapana venosa*, and *Mytilus edulis*) were also analyzed for the presence of antibiotics in their flesh (Li et al., 2012). The authors found 22 antibiotics, including quinolones, sulfonamides and macrolides, in all samples of molluscs. The analysis of seawater from the Antarctic (Hernández et al., 2019) around South Shetland Island Fildes Peninsula, where are few scientific stations from several countries, showed the presence of antibiotics in seawater. The most prevalent antibiotics were two quinolones, ciprofloxacin, and norfloxacin, at average concentrations of 0.89 μg/L and 0.75 μg/L, respectively. Three macrolides were identified: Azithromycin and clarithromycin (average concentrations close to 0.4 μg/L), and erythromycin (average concentration 0.003 μg/L). González-Alonso et al. (2017) also found antibiotics in this area. Clarithromycin, ofloxacin, sulfadiazine, sulfamethazine, trimethoprim, and sulfamethoxazole were among the most consumed pharmaceuticals at all Antarctic stations nearby the sampling area. This datum could represent a risk mainly associated with the increase in antibiotic-resistance phenomenon. Continuous delivery of antibiotics to the marine environment strongly signals that anthropogenic activities are one of the primary sources of antibiotics in marine environments. The presence of antibiotics can lead to the conclusion that pharmaceuticals’ continuous pressure on microbial communities can lead to independent ways to develop resistance against the most concentrated or most effectively eliminating substances.

### 2.2. Effect of aquacultures

Even not targeted on environment purifying, bivalve farms perfectly fulfill their role in assessing AR spreading. In contrast to finfish aquaculture, bivalve aquaculture is fed with natural phytoplankton or detritus, and no additives such as antibiotics are added during their post-larval life (Wijsman et al., 2019). Moreover, the ecological importance of bivalve molluscs as filter feeders gives them a unique position in aquaculture systems as they are used to purify water from farming in multi-trophic aquaculture systems (MTA) (Sanz-Lazaro and Sanchez-Jerez, 2020). Thus they hold a particular position in AR distribution in marine environments (Figure 1). A field study comparing traditional and MTA aquaculture of fish *Chanos chanos* revealed that the latter system can prevent the shift in microbial communities and persistence of sulphonamide-resistant phenotypes (Ying et al., 2018). In the spatiotemporal study of bacterial resistance against florfenicol in the area of intense salmon farming, the microbiota of mussel *M. chilensis* revealed a striking influence depending on the distance to the next fish farm concerning water currents (Ramírez et al., 2022). Another study pinpoints AR against antibiotics used in aquaculture but against colistin–an antibiotic of last resort against multidrug-resistant Gram-negative bacteria in human medicine in Portuguese mussel farms (Salgueiro et al., 2021).

Similarly, a broad spectrum of AR genes was found in *Ruditapes philippinarum* clam (Dahanayake et al., 2019). Despite the importance of mussels in assessment and AR clearance, the products from mussels farms may become a public health threat for consumers. Selective pressures exerted by the overuse or misuse of antibiotics in primary food production, genetically modified crops with antibiotic-resistance marker genes, microorganisms added intentionally to the food chain (probiotic or technological) with potentially transferable antimicrobial resistance genes, food processing technologies used at sub-lethal doses (e.g., alternative non-thermal treatments), and the use of biocides are among the main driving forces behind the selection and spread of antimicrobial resistance throughout the food chains (Udoekong et al., 2021).

### 2.3. Supply chains of seafood

In the supply chain, molluscs usually are subjected to several stressors during capture, depuration, storage, and transportation, including prolonged periods of air exposure and temperature fluctuation. The stressors strongly decrease the bivalves’ quality, cause some changes in the bivalves’ metabolism, and may favor the growth of bacteria, an inadvisable component of meat. According to Pan et al. (2018), depuration with UV light decreases the number of bacteria significantly but lowered temperature during the depuration process extends the number of surviving clams and lowers the observed bacteria number compared to molluscs depurated at higher temperature (2,630 vs. 1,090 CFU/ml (colony forming units). The temperature can also affect the glycogen content and pH of bivalve tissues. Unlike crustaceans and finfish, molluscs’ flesh contains an appreciable amount of glycogen. Thus, the spoilage of molluscs is primarily glycolytic (1–5% glycogen content) rather than proteolytic, which may lead to a pH decrease resulting in a lower pH than in the case of other seafood. Lower pH at later stages of spoilage may favor the growth of bacterial species preferring lower pH, like enterococci, lactobacilli, and yeasts. For this reason, animals are usually transported to the point of sale, where they undergo further processing (Kontominas et al., 2021).

The analysis of bacterial communities on flash during the storage process of Manila clams (*R. philippinarum*) confirmed that each day of storage changes the proportions of bacterial taxons significantly. Authors showed it in the example of Proteobacteriales, including the class of Gammaproteobacteria with, among other, pathogenic genera like *Vibrio, Escherichia, Pseudomonas*, and *Aeromonas.* Proteobacteria are considered typical spoilage organisms for fresh seafood (Liu et al., 2020) and, like *Aeromonas hydrophila, A. sobria* or *Shewanella putrefaciens*, *Vibrio* spp. dominate the bacterial communities in many seafoods (Madigan et al., 2014; Chen et al., 2019; Zhuang et al., 2021).

Both fresh and cooked immediately after collection, bivalves may be at risk of colonization caused by pathogenic bacteria (Ekanem, 1998; Baris Bingol et al., 2008; Ates et al., 2011). These bacteria can originate from the natural environment or result from human, animal or abiotic activities, e.g., rain washing out the organic pollution from lands into the water (Alegbeleye and Sant’Ana, 2020), wave action and rainfall (Hernandez et al., 2020; Udoekong et al., 2021). The marine ecosystem can be considered an extensive reservoir of ARB (Antibiotic Resistant Bacteria) and ARGs mainly acquired through fecal contamination of human and/or animal origin (Figure 1). Publications report the bacterial microbiota in coastal and marine ecosystems, including *E. coli, Clostridium perfringens, Bacillus cereus, S. aureus, Salmonella* spp. and others (Islam and Tanaka, 2004; Moreno Roldán et al., 2011; Grabowska et al., 2020; Verga et al., 2020; Silva dos Santos et al., 2022). Although global seafood production is growing yearly, systematic surveillance of AMR in this sector is mainly lacking or has only been at a pilot study level. One main reason for this gap is the difficulties of investigating AMR phenotypes of marine bacteria, which often have specific growth requirements, and often lack standardized antimicrobial susceptibility testing methods and interpretation criteria (Delannoy et al., 2022). Among the most frequently found bacteria in bivalve molluscs are those belonging to the *Proteobacteria* phylum (Pierce and Ward, 2019). In this phylum, there are found commensal bacteria (e.g., *Bacillus* spp., *Vibrio* spp., and *Aeromonas* spp.) and non-commensal bacteria (e.g., *Shewanella algae*). Most bacterial species’ prevalence in bivalves corresponds to the beach’s pollution degree (Silva dos Santos et al., 2022) and general water bacteriological quality (Verga et al., 2020). However, there are much more bacteria than “most interesting” human and animal pathogens. We observe bacterial genera being narrow pathogens of animals like *Francisella* spp. (Duodu et al., 2012; Charles et al., 2021; Seong et al., 2021) and species like *F. halioticida*, which is a dangerous pathogen of giant abalone (*Haliotis gigantea*) and Yesso scallops (*Mizuhopecten yessoensis*) (Charles et al., 2021). This particular species is now known as endangering the mussel cultures (Cano et al., 2022), causing economic losses and a decline in mussels. Some bacteria may also break the barrier between the animal and human-animal species and cause disease symptoms and outbreaks like *Vibrio fluvialis* (Ramamurthy et al., 2014; Onohuean et al., 2022).

## 3. The monitoring of the pathogenic bacteria in seawater and seafood

### 3.1. Institutional approach

The magnitude of antimicrobial resistance in many parts of the world has reached alarming levels and suggests that a “post-antibiotic era” may be a real possibility for the 21st century (Vidovic and Vidovic, 2020). Monitoring the pathogenic bacteria in seafood still needs more detailed and accessible programs. Table 1 presents programs that directly or indirectly refer to microbiota monitoring in the food/seafood in European countries or USA, only sometimes mentioning worldwide regulations (Food and Agriculture Organization [FAO], and World Health Organization [WHO], 2020a). The fundamental problem of most of these programs is the limited time of operation or incomplete data with a relatively small geographical coverage. Apart from those programs, there were also found regulations and directions aiming to control the quality of the seafood that included regulations of safety checks of microbiota, e.g., Europe Commission implementing regulation regarding imports of live, chilled, frozen, or processed bivalve molluscs, echinoderms, tunicates, marine gastropods for human consumption from USA (last updated 2022). Pathogenic bacteria species that were more likely reported and/or required to be reported by all sorts of programs and conventions in different countries were: *E. coli, V. vulnificus, V. parahaemolyticus*, and *V. cholerae.*

**TABLE 1:** Official seafood monitoring sanitary checks.

Food type and/or environment	Pathogenic bacteria tested during the inspection	Antibiotic-resistance (mentioned: YES/NO), if needed, a short explanation	Inspected elements/Methods	Location	Source of information
Seafood (depends on the program). Seafood in general, or bivalves	*Escherichia coli* and the *Coliform Bacteria*, *Salmonella, Shigella, Campylobacter*, *Yersinia enterocolitica*, *Vibrio*: *V. cholerae*, *V. mimicus*, *V. parahaemolyticus*, *V. vulnificus*	NO	Levels of: ● Nutrients (e.g., calcium and iron) ● Contaminants (e.g., cadmium and lead) ● Foods commonly eaten by people ● Microbiotic resistance	USA	**FDA** (Food and Drug Administration). ● Programs on microbiota: ● Total Diet studies (**TDS**) ●**NSSP** (National Shellfish Sanitation Program) ● **BAM** (Bacteriological Analytical Manual) ●**NOAA—Sea Food Inspection** Program (Fish, bivalves, and fishery products)-FDA gives certificates to industries that pass inspection
Bivalves	*V. vulnificus*, *V. parahaemolyticus*	NO		USA	National Shellfish Sanitation Program (**NSSP**) is the Federal/State cooperative program and the Interstate Shellfish Sanitation Conference (**ISSC**) for the sanitary control of bivalves recognized by the U. S. Food and Drug Administration (**FDA**)
Bivalves/ Molluscs	*Escherichia coli*	NO	● Sanitary survey (fecal contamination) ● Assess quantities of organic pollutants	Europe	Community Guide to the Principles of Good Practice for the Microbiological Classification and Monitoring of Bivalve Mollusc Production and Relaying Areas with regard to Regulation 854/2004
Seafood	pre-harvest: *Salmonella, Shigella*, *E. coli*, *V. cholerae*, *V. parahaemolyticus*, *V. vulnificus*; post-harvest *Listeria monocytogenes*, *Clostridium botulinum*, *Staphylococcus aureus*	NO		Worldwide	Code of Practice for fish and fishery products (FAO and WHO) Hazard analysis and critical control point HACCP and Defect Action Point (DAP) Analysis
Bivalves	*Salmonella* spp., *E. coli*	NO		USA	Program for Hygiene and Sanitary Control of Bivalve Molluscs (PNCMB)
All sorts of food, separate chapter for seafood	Limits for fishery products: *E. coli*	NO		Europe	COMMISSION REGULATION (EC) No 2073/2005 of 15 November 2005 on microbiological criteria for foodstuffs; COMMISSION IMPLEMENTING REGULATION (EU) 2022/158
All sorts of food	*E. coli* and *Klebsiella pneumoniae*	YES *E. coli* and *K. pneumoniae* and vancomycin resistance in *Enterococcus faecium*		Europe	**European Center for Disease Prevention and Control and World Health Organization.** Regional Office for Europe. (2022). Antimicrobial resistance surveillance in Europe 2020—2022 data. World Health Organization. Regional Office for Europe
Data from invasive isolates (blood and cerebrospinal fluid)		YES ● *Escherichia coli* ● *Klebsiella pneumoniae* ● *Pseudomonas aeruginosa* ● *Acinetobacter* species ● *Streptococcus pneumoniae* ● *Staphylococcus aureus* ● *Enterococcus fecalis* ● *Enterococcus faecium*		Europe	European Antimicrobial Resistance Surveillance Network (EARS-Net)
Monitoring of *Vibrio* in the Baltic Sea	*Vibrio cholerae*	NO		Europe	*Vibrio* growth monitoring in the Baltic Sea by ECDC https://www.ecdc.europa.eu/en/publications-data/vibrio-map-viewer https://ehp.niehs.nih.gov/doi/10.1289/EHP2198
Monitoring of zoonoses (at least from cattle, pigs and poultry)—seafood not required	*Salmonella* spp., *Campylobacter jejuni* and *Campylobacter* spp. Other to be included in monitoring: brucellosis, campylobacteriosis, echinococcosis, listeriosis, salmonellosis, trichinellosis, tuberculosis due to *Mycobacterium bovis*, verotoxigenic *E. coli*	YES		Europe	Directive 2003/99/EC of the European Parliament and of the Council of 17 November 2003 on the monitoring of zoonoses and zoonotic agents, amending Council Decision 90/424/EEC and repealing Council Directive 92/117/EEC

Nevertheless, some countries have developed programs to control seafood safety and quality (Table 1). The Food and Drug Administration (FDA) in the USA holds several large programs on microbiota presence and resistance in seafood directed explicitly at bivalves, e.g., NSSP (National Shellfish Sanitation Program run since 1925, available revisions from 2007-2019 every 2 years).^1^ This program aims to promote and improve molluscs and other shellfish sanitation. In Europe, the leading organization contributing to the collection and awareness of the need for the collection of microbiota data is European Center for Disease Prevention and Control (ECDC, run since 1988, changed its name to recent one in 2010). ECDC holds programs such as EARS-Net (collect, and compare AMR data, analyze temporal trends in Europe, and provide AMR data for policy decisions) and monitors the *Vibrio* spp. growth in the Baltic Sea during the summer seasons (since 2013) (Table 1).

### 3.2. Scientific high throughput analyses

Bacterial communities are selected and suppressed by antibiotics delivered from other natural producers—microorganisms and antibiotics produced by humans. These last ones may affect marine and freshwater bacteria and might be a selective factor for antibiotic-resistance developed by many strains individually or acquired by HGT (horizontal gene transfer), conjugation and transformation mechanisms. Previous studies have reported that ARGs might be horizontally transferred by the facilitation of class 1 integron-integrase gene (*intI1*) on urban beaches by conjugating bacterial plasmids (Jang et al., 2021).

Heavy use of antibiotics in aquaculture may highly contaminate the water bodies; hence, detecting antimicrobial residues in the water environment is also significant. Similarly, producing sewages may result in AR genes’ pollution and introduce new variants of these genes into the marine environment. The importance of this growing problem can be illustrated by the emergence of plasmid-mediated colistin-resistance among human, animal, and zoonotic pathogens (Vidovic and Vidovic, 2020). The presence of the *mcr-1* gene responsible for the resistance to colistin was observed by Valdez et al. (2022) (Table 2) in undefined *Vibrio* species and coliforms (*E. coli* and *Citrobacter freundii*) isolated from clams (*Ruditapes decussatus* and *R. philippinarum*). Samples were collected in Óbidos Lagoon (Caldas da Rainha, Portugal) in 2019, and other *R. decussatus* samples were collected in Ria Formosa (Algarve, Portugal). Antimicrobial susceptibility tests were performed by the disk diffusion method, according to the EUCAST guidelines against the antimicrobial: Colistin (10 μg), cefotaxime (30 μg), imipenem (10 μg), ciprofloxacin (5 μg), and meropenem (10 μg). The colistin-resistance gene was carried in the *Vibrio* spp. chromosomes. The *mcr-2* gene was previously found by Lei et al. (2019) in *V. parahaemolyticus* VP181, which was obtained from a shrimp sample collected from Hong Kong, China. The gene of colistin-resistance was carried on a plasmid. Cherak et al. (2021) expressed concern that antibiotic-resistance is widely distributed in the aquatic environment. The authors collected data concerning the colistin-resistance data and proved that *mcr* genes are widely distributed in many geographical regions, types of environments and many Gram-negative bacterial species. Khedher et al. (2020) published work that analyzed 64,628 genome sequences belonging to 1,050 bacterial species and had 6′651 significant positive hits indicating the presence of *mcr* genes. Among others, MCR-1, 2 and 6 reveal that the putative progenitors of these MCR variants would be bacteria belonging to *Moraxella, Enhydrobacter, Dichelobacter, Psychrobacter, Methylophilaceae, Limnobacter*, and *Vibrio* genera. It is interesting to note that all these bacteria originate from water and/or soil environments. Also, a considerable number of *mcr*-9-like sequences were identified with 3′056 hits. Most of these bacterial genera, including, *Stenotrophomonas, Vibrio, Aeromonas, Shewanella, Moraxella, Buttiauxella*, and *Salmonella*, are bacteria from environmental sources (Vidovic and Vidovic, 2020).

**TABLE 2:** Antibiotic resistance in bivalve aquaculture.

Bacteria	Farmed animal	Resistance against	Genes involved	References
*Aeromonas* spp.	*M. galloprovincialis* ^2^	Ampicillin, Aztreonam, Ceftazidime, Chloramphenicol, Ciprofloxacin, Cefotaxime, Piperacillin, Tetracycline, Ticarcillin, Tobramycin, Piperacillin/Tazobactam	*blaCTX-M-15, blaSHV-12, blaCTX-M-15, blaSHV-12, laFOX-2, blaPER-1*	Maravić et al. (2013)
*Aeromonas* spp.	*Tegillarca granosa* ^1^	Ampicillin, Piperacillin, Tetracycline, Oxytetracycline, Colistin Sulphate Erythromycin, Cephalothin, Imipenem, Ceftriaxone, Rifampicin	*aac(6*′*)-Ib, tetE, qnrB, strA-strB, qnrS, blaCTX, blaSHV, Intl 1*	Dahanayake et al. (2020)
*Campylobacter* spp.	Mussels	Ciprofloxacin, Trimethoprim-Sulfamethoxazole, Ampicillin, Cefazolin, Azithromycin, Gentamicin	*blaOXA-493*	Lozano-León et al. (2021)
*Citrobacter braakii*	*R. decussatus*	Amoxicillin, Amoxicillin + Clavulanic Acid, Cefoxitin, Chloramphenicol, Florfenicol, Oxytetracycline	*qnrB-type*	Salgueiro et al. (2021)
*C. freundii*	*C. gigas*	Amoxicillin, Ceftazidime, Cefoxitin, Florfenicol, Oxytetracycline	*qnrB44*	Salgueiro et al. (2021)
*E. coli*	Argopecten purpuratus	Florfenicol	*floR, cmlA*	Paschoal et al. (2017)
*E. coli*	*Mytilus* spp.	Amoxicillin + Clavulanic Acid, Ceftazidime, Ciprofloxacin, Chloramphenicol, Florfenicol, Flumequine, Oxytetracycline, Trimethoprim/Sulfamethoxazole	*qnrB19, blaTEM-1*	Salgueiro et al. (2021)
*E. coli*	Molluscs, not specified^1^	Ampicillin, Amoxicillin, Trimethoprim, Trimethoprim/Sulfamethoxazole, Tetracycline, Chloramphenicol, Mecillinam, Streptomycin, Nalidixic acid, Cefotaxime, Sulfamethoxazole, Ceftazidime, Gentamicin, Tobramycin, Doxycycline, Kanamycin, Norfloxacin	*blaTEM-1B, blaTEM-1C, strA-strB, blaCTX-M-14, dfrA5, dfrA17, sul1, sul2, tet(A), tet(B), tet(D), aac(3)-IId, qnrS1, dfrA14, aph(30)-Ia, catA1, aadA5, mph(A), catA1*	Grevskott et al. (2017)
*Pseudomonas. s*pp.	*Argopecten purpuratus*	Florfenicol	*floR, cmlA*	Paschoal et al. (2017)
*P. aeruginosa*	*M. galloprovincialis^2^*	Piperacillin, Piperacillin/Tazobactam, Ceftazidime, Cefotaxime, Imipenem, Meropenem, Aztreonam, Gentamicin, Tobramycin, Ciprofloxacin, Trimethoprim-Sulfamethoxazole	*bla*_*TEM–116*_, *sul1,aadA7*	Maravić et al. (2018)
*Raoultella ornithinolytica*	*Mytilus* spp.	Ciprofloxacin, Flumequine, Nalidixic acid, Oxytetracycline	*oqxAB*	Salgueiro et al. (2021)
*Salmonella enterica*	*Mussels*	Cefuroxime, Cefoxitin, Gentamicin, Tobramycin, Cephalothin, Amikacin, Ampicillin, Trimethoprim/Sulfamethoxazole, Nalidixic acid, Ciprofloxacin	*blaTEM-1B, aadA2, sul1, mph(A), dfrA12, aadA1, fosA, aph(6)-Id, aph(3”)-Ib, cmlA1, sul2, sul3, tet(A), tet(B), dfrA1, mcr-1, fosA7*	Lozano-León et al. (2021)
*Shewanella algae*	*Mytilus spp., C. gigas*	Amoxicillin, Flumequine, Oxytetracycline, Ciprofloxacin, Colistin	*qnrA3, qnrA11, qnrA12, qnrA2*	Salgueiro et al. (2021)
*Vibrio* spp.	*Tegillarca granosa*	Amikacin, Ampicillin, Cefalothin, Cefotaxime, Ceftriaxone, Colistin, Erythromycin, Imipenem, Kanamycin, Oxytetracycline, Piperacillin, Rifampicin, Streptomycin, Trimethoprim	*aac(6’)-Ib, blaCTX, aphAI-IAB, blaTEM, TetB, blaSHV, laSHV, strAB*	Dahanayake et al. (2020)
*Vibrio* spp.	Argopecten purpuratus	Florfenicol	*floR*	Paschoal et al. (2017)
*Vibrio spp.*	Clams	Chloramphenicol, tetracycline, amoxicillin, streptomycin	*cat II/tet(D), cat III*	Dubert et al. (2016)
*Vibrio* spp.	*Mytilus coruscus*	Amoxicillin, Ampicillin, Ceftriaxone, Colistin Sulfate, Erythromycin, Imipenem, Kanamycin, Cephalothin, Oxytetracycline, Piperacillin, Streptomycin	*blaCTX-M, blaTEM, blaSHV, blaOXA, IntI1, strA. B, aphA-IAB, IntI1, tetE*	Hossain et al. (2020)
*Vibrio* spp.	*Ruditapes decussatus, R. philippinarum*	Colistin	*mcr-1*	Valdez et al. (2022)
*V. alginolyticus*	*Anadara granosa*	Penicillin, Ampicillin, Vancomycin, Erythromycin	*pbp2a, blaOXA, vanB, ermB*	Shahimi et al. (2021)
*V. alginolyticus*	*Ruditapes decussatus*	Ampicillin, Tetracycline, Streptomycin, Erythromycin, Chloramphenicol, Oxolinic Acid, Gentamycin, Cotrimoxazole, Ciprofloxacin, Flumequine	*ermB, tetS*	Mechri et al. (2017)
*V. parahaemolyticus*	*Paphia undulate, Mactra veneriformis, Perna viridis* ^1^	Cephalosporin, Phenicol, Fluoroquinolone, Tetracycline, Beta-Lactam, Diaminopyrimidine, Macrolide, Nitroimidazole	*acrB, catB, SoxR, hns, acrB, qnr, crp, tet(35), tet(34), hns, acrB, SoxR, blaCARB-18, blaCARB-19, blaCARB-21, blaCARB-23, blaCARB-29, blaCARB-33, blaCARB-41, blaCARB-44, folA, macB, msbA*	Xu et al. (2022)

^1^Farmed as well as wild, ^2^Wild.

A work published in 2022 by Delannoy et al. (2022) concerned the presence of ARGs and mobile genetic elements (MGE) in the marine environment, including seafood. The team tested 268 seafood isolates of the bacterial microflora for the presence of 41 ARGs and 33 MGEs, including plasmid replicons, integrons, and insertion sequences in Gram-negative bacteria. They show the occurrence of *sul-1, ant(3″)-Ia, aph*(30)-Ia, *strA, strB, dfrA1, qnrA*, and *blaCTX-M-9* genes in *Pseudomonas* spp., *Providencia* spp., *Klebsiella* spp., *Proteus* spp., and *Shewanella* spp. They found that the occurrence of MGE may be associated with the seafood type and the environmental, farming, and harvest conditions. Their results corroborate the hypothesis that the incidence of antimicrobial-resistant bacteria (ARB) and ARG decrease with increasing distance from potential sources of fecal contamination. By high throughput real-time PCR system and array designed for antimicrobial resistance markers, they confirmed the presence of some ARGs like *blaCMX-M-9* and *aph*(30)*-Ia* in *Klebsiella* spp., *qnrA* in *Shewanella* spp., and *sul1* and *ant(3″)-Ia* in *Pseudomonas* spp. In 2019, Fresia et al. (2019) analyzed microbial communities harboring ARG and their characteristics. They collected samples from 20 localities around Montevideo (Uruguay). They found that ARGs covering resistance to sulfonamides, beta-lactams, aminoglycosides, phenicols, macrolides, and streptogramins were more prevalent in plasmids than in bacterial chromosomes. At the same time, ARGs covering resistance to tetracyclines, lincosamides, fluoroquinolones, and elfamycins were more frequently encoded in chromosomes. In 61% of identified integrons, Fresia et al. (2019) found cassette ARGs with conserved *attC* sites. These cassette genes primarily coded for multi-drug efflux pumps, but authors also found genes coding for carbapenemases (OXA family), GES extended-spectrum beta-lactamases (ESBLs), and aadA1 aminoglycoside nucleotidyltransferases, chloramphenicol acetyltransferases, and aac6-Ib amikacin resistance. This fact may support a hypothesis that ARGs carried by bacteria mostly present in sewages are potentially one of the most significant anthropogenic problems supporting the spreading of microbial resistance into marine bacterial species. Paschoal et al. (2017) indirectly confirmed it by finding carbapenemase genes in *Aeromonas, Pseudomonas, Enterococcus, Klebsiella*, and other genera isolated from recreational coastal waters, gene variants such as *blaGES-16, blaIMP-16, blaOXA-370*, and *blaOXA-143* which were so far reported only (or predominantly) in clinical isolates from Brazil.

In the Pacific Ocean and the Antarctic, similar research done by Jang et al. (2022) displayed the presence of ARGs in transects including the West Sea of Korea, the Philippine Sea, the Western Equatorial Pacific Ocean, the Tasman Sea, the New Zealand sector of the Southern Ocean, and the Ross Sea. They found tetracycline resistance genes (*tetA, tetB, tetBP, tetD*, and *tetZ*), sulfonamide-resistance gene (*sul1*), one macrolide resistance gene (*ermB*), one β-lactam resistance gene (*blaTEM*), one quinolone-resistance gene (*qnrD*), and one multi-antibiotic resistance gene (*oqxA*). Overall, ARG abundance in the Southern Ocean was lower than in the Western Pacific Ocean but increased toward Antarctica. This pattern may be affected by human activities in the research stations (e.g., residence for research purposes or tourism) and incomplete wastewater treatment in the Antarctic environment.

Probably the part of bacteria carrying the ARGs may be finally deposited in all filtering organisms living in local seawaters, but even most disturbing is the knowledge that the geographical distribution of these genes seems to be continuous and unlimited. This fact especially concerns human and animal pathogens and emerging species with the potential to outbreak in human populations. It must be noted that the occurrence of antimicrobial-resistant bacterial species is not always a signal of pollution. Many species exhibit natural antibiotic-resistance, which is also expected in the natural marine environment. Miranda et al. (2013, 2015) presented the role of scallops hatcheries as a reservoir of antibiotic-resistant bacterial species. According to the presented data, the resistance of bacteria occurred even when no antibiotics were used.

## 4. The most popular gram-negative pathogenic bacteria in molluscs

Gram-negative bacteria are the focus of the World Health Organization (WHO). In 2017, the World Health Organization (WHO) published a list of global priority pathogens (GPP)—12 species of bacteria with critical, high, and medium antibiotic-resistance (AR), where 9 out of the 12 pathogens were gram-negative (Asokan et al., 2019). The outer membrane of Gram-negative microorganisms may be a significant barrier that protects toxic compounds, which include antibiotics and host innate immune molecules such as cationic antimicrobial peptides. At the same time, antibiotic-resistance mechanisms in Gram-negative bacteria may involve the acquisition of enzymes that modify or destroy antibiotics. The mechanisms may also include acquiring enzymes that alter bacterial antibiotic targets, such as lipid A-modifying enzymes conferring resistance to colistin and acquiring mutations in bacterial targets, such as topoisomerases, ribosomes, and penicillin-binding proteins, and outer membrane porins that alter antibiotic efficacy or uptake (Miller, 2016).

### 4.1. *Pseudomonas* spp.

Members of the genus *Pseudomonas*, which belongs to the γ-Proteobacteria, can colonize diverse habitats. The main fraction of *Pseudomonas* species described so far was assigned to terrestrial habitats (Manwar et al., 2004; Romanenko et al., 2005, 2008, 2015); however, there are also reports of species isolated from marine environments, e.g., *P. glareae* (Romanenko et al., 2015) or *P. marincola* (Romanenko et al., 2008), *P. pachastrellae* (Romanenko et al., 2005), and *P. aeruginosa* (Manwar et al., 2004). The species belonging to *Pseudomonas*, widespread in scallop hatcheries (Miranda et al., 2013, 2015), constitutes a significant group of antimicrobial-resistant species. Ramírez et al. (2022) published similar results for *Mytilus* species reared in different years and distances from salmon farms. Authors showed that *Mytilus* spp. microbiota differs depending on the time and proximity to salmon farms. They also showed that *Pseudomonas* spp. represent the majority of florfenicol-resistant isolates (89% in 2018 and 78% in 2019). The number of resistant isolates compared with several other species that acquired the resistance for florfenicol may suggest that *Pseudomonas* spp. are quick at obtaining the resistance to florfenicol and/or colonizing new hosts as members of the first wave of colonizing bacterial species. This hypothesis is supported by data in described publication showing the relative abundance of *Pseudomonas* spp. per location was <1% but represented the most resistant isolates. In the mentioned publication, along with *Pseudomonas* spp., only two more genera (*Microbacterium* spp. and *Psychrobacter* spp.) were represented by florfenicol-resistant isolates. Following the year, the number of florfenicol-resistant genera increased to four (*Acinetobacter* spp., *Alcaligenes* spp., *Psychrobacter* spp., and *Serratia* spp.).

*Pseudomonas aeruginosa* is the third most pathogenic Gram-negative bacteria capable of causing severe diseases (Nolan and Behrends, 2021). This species may be a serious issue in patients with ventilator-associated pneumonia, urinary/peritoneal dialysis catheter infections, bacterial keratitis, cystic fibrosis, and burn wounds (Nolan and Behrends, 2021). *P. aeruginosa* causes around 13–19% of hospital-acquired infections (HAIs) in the USA (Raman et al., 2018). This species is primarily a waterborne pathogen, thriving in moist habitats and water resources like outlets of sewage treatment plants, from where it can be further spread to the marine environment (Luczkiewicz et al., 2015). In 1975, Denis (1975) observed *P. aeruginosa* in over 70% of mussels from the Marennes–Oleron Basin (France). Maravić et al. (2018) analyzed *Pseudomonas* spp. by combining PCR detection of acquired genes and resistance-nodulation-cell division (RND) efflux, studying multifactorial resistant traits of 108 *P. aeruginosa* isolates recovered from wild-growing Mediterranean mussels (*Mytilus galloprovincialis*) in Croatia. The multi-drug resistance to at least three pharmaceuticals from three different classes was detected in 17 isolates (15.7%). The most common (8.3% of isolates) profile included resistance to piperacillin (PIP), piperacillin-tazobactam (TZP), ceftazidime (CAZ), cefotaxime (CTX), and aztreonam (ATM). These substances represented penicillins, β-lactam/β-lactamase inhibitor combinations, cephems, and monobactams. Generally, 23.14% of isolates were resistant to at least one pharmaceutical. Also, Udoekong et al. (2021) examined 117 clams, 88 oysters, and 136 periwinkles. The authors found *P. aeruginosa* strain JB2 in 7.4% of tested molluscs, *P. aeruginosa* strain N15-01092 in 2.2%. Other species of *Pseudomonas* found in examined samples were *P. xiamenensis* (3.7%) and *P. anguillisceptica* 4,029 (2.2%). Generally, 44 isolates of *Pseudomonas* species (100%) were resistant to ceftriaxone, cefpodoxime, ceftazidime, cefepime, imipenem, and chloramphenicol. Unfortunately, the authors did not check ARGs’ presence, but the appropriate genes like *cmlA* and integrons were undoubtedly present. The literature review did not provide enough data to compare the antibiotic-resistance of environmental isolates of *P. aeruginosa* and other species with terrestrial isolates, although the *Pseudomonas* clade probably has a water environment origin. Generally, Laborda et al. (2021) considered *P. aeruginosa* as the species that succeeded in colonizing multiple hosts by relying on its large genome, and the species’ virulence and resistance determinants evolved in natural ecosystems.

### 4.2. *Aeromonas* spp.

Bacteria belonging to the genus *Aeromonas* are ubiquitous in marine environments, and *Aeromonas* spp. has held the title “emerging foodborne pathogen” (Janda and Abbott, 2010; Chuang et al., 2011; Abdel-Latif and Khafaga, 2020; Fernández-Bravo and Figueras, 2020; de Silva et al., 2021). *Aeromonas* spp. is a Gram-negative, non-spore-forming rod-shaped, facultatively anaerobic bacteria easily found in aquatic environments. Members of the genus *Aeromonas* are potential pathogens to many hosts, such as fish, amphibians, reptiles, mammals, and humans (Janda and Abbott, 2010; Fernández-Bravo and Figueras, 2020). *Aeromonas* spp. may cause various human infections, such as gastroenteritis, soft tissue infection, septicemia, hepatobiliary tract infections and occasionally pleuropulmonary infections, indwelling device-related infections, meningitis, peritonitis, and haemolytic uremic syndrome (Chuang et al., 2011). Among *Aeromonas* species that cause fish diseases, *A. hydrophila* has been identified as one of the most dangerous pathogens causing mortality outbreaks in diverse cultured fish species (Abdel-Latif and Khafaga, 2020). Among pathogens associated with human diseases, *A. caviae, A. veronii*, and *A. hydrophila* biovar *sobria* were isolated from mussels, scallops, and oysters (de Silva et al., 2021). These species *Aeromonas* spp. can grow relatively uninhibited in food during refrigeration under a broad range of pH and NaCl concentrations and in various packaging atmospheres. Given its high prevalence in seafood included in many RTE (Ready-To-Eat) seafood meals, the significance of *Aeromonas* spp. as a potential foodborne pathogen and a food spoilage organism increases. Strains of several *Aeromonas* species have shown spoilage potential by producing spoilage-associated metabolites in various seafood products (Hoel et al., 2019). Another point is the presence of genes encoding exotoxins, such as haemolysin (*hlyA*), aerolysin (*aerA*), cytotoxic heat-labile enterotoxin (*act*), cytotonic heat-labile enterotoxin (*alt*), cytotonic heat-stable enterotoxin (*ast*), and Shiga-like toxins (*stx*). Mentioned exotoxins are well-known virulence factors related to clinical symptoms during *Aeromonas* infection. A problem is deepened by the simultaneous harboring of AR genes. Maravić et al. (2013) found isolates of *Aeromonas* spp. co-producing *blaCTX-M-15, blaSHV-12, blaPER-1*, and *blaFOX-2* in Mediterranean mussels (*Mytilus galloprovincialis*) from Croatia (Table 2). Generally, *A. caviae*, and *A. hydrophila* displayed full phenotypic resistance to ampicillin; 86% were resistant to cefotaxime, 68% to ticarcillin, and 63% to aztreonam. Hossain and Heo (2022) found in *Mytilus coruscus* from South Korea, 33 *Aeromonas* species are carrying *blaTEM* (27.3%), *blaSHV* (24.2%), *blaCTX-M* (24.2%), *tetB* (18.2%), *tetE* (15.2%), and *intI1* genes (27.3%). Their results suggest that mussels can serve as a reservoir of multi-drug resistant *Aeromonas* spp. Manila clams (*R. philippinarum*) purchased on the market in South Korea also contained *A. hydrophila, A. media, A. veronii, A. allosaccharophila*, and *A. caviae* resistant to various antibiotics (Sanz-Lazaro and Sanchez-Jerez, 2020). Isolates of *Aeromonas* spp. harbored seven resistance genes (*blaTEM, blaSHV, blaCTX-M, tetB, tetE, qnrS*, and *StrA-strB*) and class 1 integron [*intl1, aac(6’)-Ib*] in various combinations. The most universal was the β-lactamase-resistance complex of genes (*bla*) (Table 2) (Dahanayake et al., 2019).

### 4.3. *Vibrio* spp.

Besides the prevalent pathogens from anthropogenic and animal pollution, marine bivalves can be colonized by *Vibrio* spp. This group of bacteria consists of species living in seawater and being pathogens both to bivalves and humans. In marine environments, metazoans constitute habitats for bacteria of the Vibrionaceae family (vibrios). These γ-proteobacteria are ubiquitous in marine and brackish environments representing one of the most abundant culturable fractions of the marine microbial community (Ceccarelli et al., 2019). The most known species are *V. cholerae, V. vulnificus, V. parahaemolyticus*, and *V. alginolyticus*, but other species are also recognized as associated with food consumption. *V. vulnificus* and *V. parahaemolyticus* are seasonally present in molluscs (Lorenzoni et al., 2021) *V. fluvialis, V. mimicus, V. metschnikovii, V. metoecus*, and *V. furnissii* are related to foodborne diseases (Ceccarelli et al., 2019). Among these vibrios, *V. fluvialis* is an emerging human pathogen (Ramamurthy et al., 2014) related to diarrhoeal outbreaks. Other pathogenic species are *V. cincinnatensis* and *V. carchariae*, whose significance as human pathogens remains determined as agents causing several types of vibriosis. About 13 species of Vibrionaceae have been reported to cause several human diseases (Hou et al., 2011; Ramamurthy et al., 2014; Cheng et al., 2015). At the same time, *Vibrio* spp. frequently coexists with *Pseudomonas* spp. and *Aeromonas* spp. species, which are another group of potential human pathogens. In Benin City (Nigeria), Igbinosa (2016) found isolates of *V. fluvialis, V. vulnificus, V. parahaemolyticus* and unidentified *Vibrio* spp. in four water ponds by collecting fish and water samples. These isolates were resistant to β-lactams, macrolides, quinolones, norfloxacin, sulphonamides, nitrofurantoin, aminoglycoside, tetracyclines, and phenicol derivatives. Isolates represented a broad spectrum of resistance, from 0% of isolates resistant to ciprofloxacin to 100% resistant to tetracycline. There is no reason to exclude the possibility that vibrios cannot infect the edible bivalves in the water. Many species of cultured or wild molluscs accompany fish aquacultures serving as potential filtration systems or just as free-living animals, settling the environment rich in food particles. According to Vu et al. (2018), the distribution of *Vibrio* spp. in different species (blue mussels, venus clams, razor shells, cockles, and white leg shrimps) from retail food found in Berlin included 40.8% of blue mussels, 100% of cockles, and 93.3% of venus clams and 27.3% of razor shells. Similar results were published by Roque et al. (2009) in the case of four species of cultured molluscs on the Spanish coast (*Mytilus galloprovincialis*, *Crassostrea gigas*, *Ruditapes decussatus*, and *Ruditapes philippinarum*). In 4.08% of samples, Obaidat et al. (2017) found *V. parahaemolyticus* isolates resistant to chloramphenicol and cefotaxime in seafood from Yemen, India and Egypt. Besides this, 100% of isolates were resistant to colistin sulfate, kanamycin, and neomycin. In hard-shelled mussel (*Mytilus coruscus*) and tiger shrimp (*Penaeus monodon*) purchased in South Korea, Ashrafudoulla et al. (2019) found isolates of *V. parahaemolyticus* resistant to erythromycin, vancomycin, kanamycin, ciprofloxacin, clindamycin, and other antibiotics. In Poland (Lopatek et al., 2015) found that 17.5% of 400 tested samples of mussels, clams, oysters, and scallops were positive for the presence of *V. parahaemolyticus*. Among positive isolates, the authors found resistance to β-lactams (56%), ciprofloxacin (1.6%), streptomycin (45%), and gentamicin (7%). The food represented by bivalves is not present in the Baltic Sea, and all specimens were imported from the Netherlands, Italy, Norway and France. Dubert et al. (2016) noted that chloramphenicol had been banned in food animals, including aquaculture, because it has been associated with aplastic anemia. However, the authors noticed that experimental hatchery aquacultures still use this antibiotic due to its broad antimicrobial spectra. They analyzed samples from The Centro de Investigacións Mariñas (Spain), where seven species of clams were produced, and found that among 121 isolates of *Vibrio* spp. representing *V. hemicentroti, V. splendidus, V. kanaloae, V. neocaledonicus*, and *V. jasicida*, of non-human-pathogenic bacteria, 19 were resistant to chloramphenicol (CHL), tetracycline (TET) and amoxicillin (AMX), and nine were resistant to chloramphenicol (CHL) and streptomycin (STR). In 28 isolates of AGR genes, they confirmed the presence of the gene responsible for the resistance to chloramphenicol. Moreover, they demonstrated that antibiotic-resistance genes could be transferred to *E. coli* and other marine bacteria from *Vibrio* spp. In light of information about the possibility of transferring the ARGs among bacterial clades (Ellabaan et al., 2021), it can be highly possible that marine vibrios can serve as a potential reservoir for antibiotic-resistance genes (Table 2) for many emerging bacterial species. Besides being the reservoir of AR, a secondary problem generated by this genus is the number of exotoxins secreted by vibrios. Even bacteria from this purely marine genus can produce exotoxins (ciliostatic factors and hemolysins), which cause deciliation and injure tissues. According to West (West, 1989), the increase of *Vibrio* spp. number (up to an infective dose) can occur as water temperatures rise seasonally, which facilitates the growth and rising concentration of bacteria on/in higher animals, such as chitinous plankton, or accumulation of the bacteria by shellfish and other seafood. Pathogenic *Vibrio* species must elaborate a series of virulence factors to elicit human disease. Activities predisposing to diarrhoeal and extraintestinal infections include ingesting seafood and occupational or recreational exposure to natural aquatic environments, especially warm ones with temperatures rising above 20°C. Iwamoto et al. (2010), analyzing *V. parahaemolyticus* and *V. vulnificus* outbreaks, noticed that cases of *Vibrio* spp. infections have a marked seasonal distribution. Most occur during summer and early fall, corresponding to the period of warmer temperatures. In Japan, *V. parahaemolyticus* is one of the leading causes of foodborne gastroenteritis; annually, 500–800 outbreaks affecting more than 10,000 people are reported (Food and Agriculture Organization [FAO], and World Health Organization [WHO], 2020b). According to FDA data, between 1973 and 1991, all occasional cholera cases related to raw or undercooked shellfish consumption were associated with a non-O1 biotype of *V. cholerae* (Kaysner et al., 2012).

### 4.4. *Shewanella* spp.

*Shewanella* is the sole genus belonging to the family of marine bacteria Shewanellaceae. As far as four species of this genus are considered dangerous for humans: *S. algae, S. putrefaciens, S. haliotis*, and *S. xiamenensis* (Vignier et al., 2013; Cimmino et al., 2015; Tena et al., 2016; Huang et al., 2018). These species may cause bacteremia, Fournier’s gangrene, empyema, pneumonia, and intracranial infections (Yousfi et al., 2017). This family is represented by widespread species and extremophiles living in habitats with a broad range of temperatures and high pressures (Abboud et al., 2005; Lemaire et al., 2020). For example, *S. loihica* from thermal vents can grow at 4–42°C. The taxon includes species isolated from around all oceans, including Antarctic subglacial and arctic marine waters, tropical coastlines, deep oceanic trenches, and various fish and shellfish. Their survival in various ecological niches is due to their impressive physiological and respiratory versatility (Lemaire et al., 2020). Some strains have been isolated in particular geographic areas, suggesting a putative specificity, while other isolates were found in many distant zones. One of the most spread species, often related to seafood, is *S. putrefaciens*, the species responsible for food spoilage and disease outbreaks (Liu et al., 2006; To et al., 2010; Janda and Abbott, 2014). Many consumable products have yielded *Shewanella* species upon laboratory analysis. Among others, the analysis of oyster samples from Delaware Bay showed that concentrations of *Shewanella* (presumptive) in oyster meats ranged from non-detectable levels to ≥400 CFU/g per sample (Richards et al., 2008).

Members of this genus are generally susceptible to third- and fourth-generation cephalosporins, carbapenems, β-lactamase inhibitor combinations, aminoglycosides, chloramphenicol, erythromycin, aztreonam, and quinolones (Table 2) (Kang et al., 2013; Cimmino et al., 2015; Zago et al., 2020).

Zago et al. (2020) checked resistance against antibiotics of *Shewanella algae*. The authors collected water samples along the Veneto coastline of the Adriatic Sea, Lugi di Varano (Foggia) and the open sea. They found isolates of *S. algae* resistant to β-lactams (*blaOXA-55-LIKE, ampC*), tetracyclines (*tetR, tet34*, and *tet35*), fluoroquinolones (*qnrA3, qnrA7, mepAB*—multi-drug efflux pump, *mfpA—qnr* homolog, *emrD*- multi-drug efflux pump, and *mtdN*—multi-drug resistance protein), polymyxin (*eptA*), chloramphenicol (*cat*). Also, Delannoy et al. (2022) found *Shewanella* spp. (*S. indica, S, haliotis, S. algae*, and unidentified *Shewanella* spp.) isolates from shellfish resistant to quinolone and possessing the *qnrA* gene on a plasmid. In Asian hard clams (*M. lusoria*), Lee et al. (2019) found isolates identified as *S. algae* SYT4 harboring β-lactamase encoding *blaOXA-55* responsible for carbapenem resistance. Authors suggested that environmental *S. algae* could be a potential class D β-lactamase gene source.

Besides *blaOXA-55*, authors also identified the *dfrA3* gene associated with resistance to sulfonamide-trimethoprim and genes responsible for resistance to fluoroquinolone (*mfd, qnrA3*). Due to the high zoonotic potential and wide distribution, *Shewanella* spp. are found practically in most seafood and marine environments. The characteristics of their genomes (Nuñez et al., 2022) may suggest that this genus can intensively transfer genetic information, including AR genes. Nuñez et al. (2022) found 52% of analyzed genomes of *Shewanella* spp. with conservative integrase genes (*intI*). They showed that *Shewanella* spp. could acquire and disseminate integrase genes that may lead to the emergence of novel mobile integrons. The substantial diversity of gene cassettes found in the variable regions and their association with MGEs may imply the host’s constant evolution and adaptation. This relation responds to environmental niche changes and the composition of each microbial community. Furthermore, it must be considered that antimicrobial-resistance gene cassettes found in hospital settings can be recruited from environmental chromosomal integrons. The above characteristic may suggest the solid potential ability to adapt to new hosts and new environments, and the plasticity of that genus fully justifies treating this species as an emerging organism most dangerous to humans and animals.

### 4.5. *Escherichia coli* and coliforms

*Escherichia coli* is one of the most popular and widespread bacteria in seawater, as it is a significant member of the bacterial microbiota community in the gut of many animals and humans (Michalska et al., 2021). There is a possibility of transmission of pathogenic *E. coli* strains to the seas from land animal feces and human feces, especially during periods of intense rainfall (Lunestad et al., 2016). Consumption of raw or inadequately cooked mussels poses a potential risk for consumers because of *E. coli* infections (Feng et al., 2020). According to the current EU regulations [Regulation (EU) 2017/625 of the European Parliament and of the Council, 2017], mussel sampling sites must be classified according to their suitability for microbiological and chemical water quality (De Witte et al., 2014). The fecal indicator organism (FIO) *E. coli* is frequently used as a general indicator of sewage contamination and for evaluating the success of mollusc cleaning (depuration) processes. Depending on the content of *E. coli* in mussels, all localities should be affiliated based on Most Probable Numbers (MPN) as Class A (<230 MPN *E. coli*/100 g mussel), B (<4,600 MPN *E. coli*/100 g mussel), or C (4,600–46,000 MPN *E. coli*/100 g mussel) areas (Grevskott et al., 2016). Most of the research on the effectiveness of depuration has been conducted using oysters, as these are usually eaten raw. In 2019, Martin et al. (2019) found Shiga-toxin-producing *E. coli* (STEC) in Norwegian bivalves (1.1%). This percentage in the work of other authors was higher in shellfish, ranging from 3.5% in France (Gourmelon et al., 2006) to 6.1% in Morocco (Bennani et al., 2011). The relatively low isolation rate of STEC strains does not exclude the presence of *stx*-carrying bacteriophages in the samples, which pose a threat by playing an essential role in the evolution of STEC strains but also can transfer *stx* genes from non-pathogenic strains *E. coli* (Krüger and Lucchesi, 2015).

The multi-drug resistance of *E. coli* has become a worrying problem (Table 2). *E. coli* is internally susceptible to almost all clinically relevant antimicrobials (the ability to accumulate resistance genes mainly through horizontal gene transfer). The most complicated mechanisms in *E. coli* are the acquisition of genes encoding broad spectrum β-lactamases (broad-spectrum cephalosporin resistance), carbapenemases (carbapenem resistance), resistance to (aminoglycosides), plasmid-mediated quinolone resistance (PMQR), and *mcr* genes(conferring resistance to polymyxins) (Poirel et al., 2018).

## 5. The most popular gram-positive pathogenic bacteria in molluscs

According to Zannella et al. (2017), only a few Gram-positive bacteria cause diseases in bivalves, and the main genus is *Nocardia*. Only single species were reported as potentially dangerous for humans, but bivalves can also host non-marine pathogens causing severe healthcare problems.

### 5.1. *Nocardia* spp.

Of all Gram-positive bacteria characteristics for bivalves, one is definitively typical for the marine environment but may be pathogenic to humans. The *Nocardia* genus is a prevalent member of the molluscs microbiota. This genus can be responsible for a broad spectrum of human diseases. Species like *Nocardia nova, N. farcinica, N. cyriacigeorgica, N. brasiliensis*, and *N. abscessus* are responsible for such cases as invasive pulmonary infection, disseminated infection, or brain abscess; 20% present as cellulitis. From the marine environment, the most known species is *N. crassostrea*, responsible for *Pacific oyster* nocardiosis observed in North America from the Strait of Georgia, British Columbia, to California and Japan (Matsushima Bay). Carella et al. (2013) observed this disease in Mediterranean bivalves first time. In 2013 (Taj-Aldeen et al., 2013) and 2016 (Igbaseimokumo et al., 2016), two outbreaks of *N. crassostrea* were observed in immunocompromised patients independently. No comprehensive information about genes responsible for antibiotic-resistance or antibiogram analysis was available. Igbaseimokumo et al. (2016) added information about antibiogram results. *N. crassostrea*, in this analysis, was sensitive to beta-lactams, including carbapenems but was resistant to lincosamides (clindamycin), vancomycin, and teicoplanin. This observation suggests the constitutive mechanism of defense against mentioned groups of antibiotics if the environmental origin of *N. crassostrea* is considered. In the cases described by Taj-Aldeen et al. (2013), the authors also checked the antibiotics-resistance of *N. crassostrea* and found one isolate resistant to linezolid (oxazolidinones group) and moxifloxacin (fluoroquinolones group). Still, they found the tested strain’s sensitivity to beta-lactamases (including cefotaxime and imipenem) and aminoglycosides (amikacin).

### 5.2. *Clostridium difficile*

Symptoms of botulism include fatigue, dizziness, double vision, progressive difficulties in speaking and swallowing, shortness of breath, and muscle weakness (Aberoumand, 2010). Most seafood-related botulism is associated with inadequate processing, temperatures or maintenance (National Advisory Committee on Microbiological Criteria for Foods [NACMCF], 2008; Hudson and Lake, 2012). The occurrence of another *Clostridium* sp. in shellfish, *C. difficile*, raises concern because spores can survive the cooking temperatures given that shellfish is often consumed poorly cooked or raw. For example, *C. difficile* has been isolated from edible bivalve molluscs in Spain and Italy (Agnoletti et al., 2019; Candel-Pérez et al., 2020). *C. difficile* collected from 702 molluscs harvested in the North Adriatic Sea (Agnoletti et al., 2019) were detected in 16.9% of all bivalves specimens. Isolates showed microbiological resistance against clindamycin (17%), erythromycin (23%), rifampicin (8.8%), and moxifloxacin (10.6%). All isolates were susceptible to metronidazole, and one showed MIC> ECOFF for vancomycin. *C. difficile* strains showed a wide variety in PCR-ribotypes, most already detected in other animals or known as highly virulent and epidemic in humans. In Spain, Candel-Pérez et al. (2020) analyzed 129 mollusc samples from different fishmongers and grocery stores in Murcia. *C. difficile* was isolated from 8.53% (11 specimens per total 129) of the investigated molluscs. Four *C. difficile* isolates harbored genes for producing toxins A and B. Examining mussels for antibiotic-resistant strains of *C. difficile* should be another essential aim of seafood monitoring.

## 6. Conclusion

Consumers’ growing interest in seafood, including mussels, should be determined by constantly monitoring microbiological hazards associated with these products. Such a network should include a system of bacterial monitoring in selected geographic locations dependent on seafood production centers. Another activity should supervise the distribution of genes encoding resistance to antibiotics independently on bacterial species. The sensitivity and selectivity of modern analysis methods should also be propagated to monitor seafood for the presence of the most emerging bacterial species, such as *Pseudomonas* spp. and *Vibrio* spp. The complete data concerning AR come from seawater genomic analyses (Paschoal et al., 2017; Lei et al., 2019; Khedher et al., 2020; Cherak et al., 2021), depicting the complete analysis of genes involved in AR (Table 2).

A unified global program of antibiotic treatment recommendations and monitoring of antibiotic use in aquacultures should be created to limit the spread of mobile genetic elements coding AR. Such a program could be helpful if synchronized with environmental and food monitoring.

On a global scale, disease outbreaks have increased over the last decades in the marine environment (Baker et al., 2022). Although the definitive causes for this increase are uncertain, ocean warming (Pinsky et al., 2019; Albano et al., 2021; Pepi and Focardi, 2021) and marine pathogen emergence, reemergence or evolution leading to the colonization of new niches, including humans, appear to be critical driving forces. For example, bacteria preferring subtropical and tropical seawater like *V. vulnificus* extended their distribution to new marine areas like the Baltic Sea (Kurpas et al., 2021).

The danger for humans and marine organisms includes harmful bacterial infections and the threat from toxins produced even by harmless marine bacteria due to massive contact with land-sourced bacteria and horizontal gene exchange. In the long-term, acquiring new AR genes from different bacterial species can be dangerous for humans and endangers seafood production by limiting the efficacy of antibiotics used to protect the aquacultures. Food quality should be monitored carefully to prevent the development of bacterial resistome in the marine environment and outbreaks of super-resistant bacteria. Some public information provided to clarify standards of molluscs’ freshness rating and procedures of seafood preparation should also be present.

## Author contributions

AgK designed the form of the manuscript, wrote the text except for tables and subsections 3.1, 4.5, and 5.2, and prepared a graphic. AlK, TK, and KG-B wrote the text in the subsections mentioned above, and AlK and TK collected the data and synthesized them into tables. AgK, KG-B, and NW-K edited manuscript. AgK and NW-K prepared the literature in the present review and edited the manuscript. KS and KZ supervised the process of creation and edited the discussion. All authors contributed to the article and approved the submitted version.

## Footnotes

1
https://www.fda.gov/food/federalstate-food-programs/national-shellfish-sanitation-program-nssp
